# Medical Society

**Published:** 1897-01-23

**Authors:** 


### MEDICAL SOCIETY.

A paper was read by Mr. Bland Sutton at the last meeting on the subject of "Wandering Spleen." In the investigation of abdominal, and even of pelvic tumours, it is important always to bear in mind the extent to which a loosely-attached spleen may wander. It is difficult to conceive that a viscus like the spleen, suspended by peritoneum to the under surface of the diaphragm, connected to the great *cul-de-sac* of the stomach by means of the gastro-splenic omentum, and by its blood-vessels brought into intimate bondage with the pancreas, could so elongate its attachments as to reach the fundus of the virgin uterus. But such is the case. Occasionally it will occupy the normal position, and remain for days retired under cover of the left costal arch; then suddenly it slips out and becomes obvious to the patient in the umbilical or iliac regions; then it will descend into the pelvis, where its presence may be determined only by deep pressure of the fingers over the hypogastric region, or by vaginal examination.

It is noteworthy that wandering spleens are almost, but not exclusively, confined to women, and that in the majority of cases these women have had children. As a result of its long pedicle, and the consequent interference with the circulation of blood through it, a wandering spleen may be affected with engorgement, by which its size may be made to vary very rapidly. By axial rotation of the pedicle the veins may be so compressed that the organ may appear ready to burst with blood; or on the other hand atrophy may result from occlusion of the artery.

Wandering spleens have been confounded with many kinds of abdominal tumours, such as ovarian cysts, renal tumours, malignant tumours of the omentum, moveable kidneys, and uterine myomata. The tendency of such spleens is to sink down into the pelvis, where their weight may double up the uterus, causing retroflexion, or may cause complete prolapse of the uterus, vagina, and bladder. When in this situation their firmness and shape, combined with their tendency to cause disturbance of the menstruation, have caused such splenic tumours in many instances to be mistaken for uterine myomata. On the other hand, it must be borne in mind that ovarian and uterine tumours may closely simulate wandering spleens.

Mr. Bland Sutton considered that splenectomy for wandering spleen was not only justifiable, but was a highly successful operation, the rate of recovery being equal to that of ovariotomy in skilled hands. Attempts had been made to secure the spleen in the left hypochondrium by means similar to those adopted for moveable kidney, but he thought results showed clearly that the patients ran much greater risks by such proceedings than by having their spleens removed.

